# Characterization of Digestive Enzymes of Bruchid Parasitoids–Initial Steps for Environmental Risk Assessment of Genetically Modified Legumes

**DOI:** 10.1371/journal.pone.0036862

**Published:** 2012-05-16

**Authors:** Fernando Álvarez-Alfageme, Christoph Lüthi, Jörg Romeis

**Affiliations:** Agroscope Reckenholz-Tänikon Research Station ART, Zurich, Switzerland; U. Kentucky, United States of America

## Abstract

Genetically modified (GM) legumes expressing the α-amylase inhibitor 1 (αAI-1) from *Phaseolus vulgaris* L. or cysteine protease inhibitors are resistant to several bruchid pests (Coleoptera: Chrysomelidae). In addition, the combination of plant resistance factors together with hymenopteran parasitoids can substantially increase the bruchid control provided by the resistance alone. If the strategy of combining a bruchid-resistant GM legume and biological control is to be effective, the insecticidal trait must not adversely affect bruchid antagonists. The environmental risk assessment of such GM legumes includes the characterization of the targeted enzymes in the beneficial species and the assessment of the *in vitro* susceptibility to the resistance factor. The digestive physiology of bruchid parasitoids remain relatively unknown, and their susceptibility to αAI-1 has never been investigated. We have detected α-amylase and serine protease activities in all five bruchid parasitoid species tested. Thus, the deployment of GM legumes expressing cysteine protease inhibitors to control bruchids should be compatible with the use of parasitoids. *In vitro* inhibition studies showed that sensitivity of α-amylase activity to αAI-1 in the parasitoids was comparable to that in the target species. Direct feeding assays revealed that harmful effects of α-amylase inhibitors on bruchid parasitoids cannot be discounted and need further evaluation.

## Introduction

Grain legumes, also known as pulses or food legumes, are mainly cultivated in developing countries, where they are essential for nutrition. Pulses represent a source of income and livestock feed and meet the requirements of small-scale, low-income farmers in developing countries [1]. Grain legumes are commonly stored over extended periods to ensure supplies of household food and seed for sowing [2]. Several coleopteran and lepidopteran pests are responsible for extensive losses to stored grain legumes because these pests develop and reproduce rapidly, completing multiple generations in the storage. In addition, insect pests increase the temperature and humidity of the stored pulses, which increases grain respiration and thereby reduces grain quantity and quality [3]. The average grain-weight loss for pulses due to insect pests is 20% [4], although it can be up to 100% and is generally much higher than the loss caused by rodents, microorganisms, and other pests [2]. Larvae of several *Acanthoscelides*, *Zabrotes*, and *Callosobruchus* spp. (Coleoptera: Chrysomelidae) are among the most important insect pests of pulses worldwide.

Many insects, especially those like bruchids that feed on starchy seeds, depend on α-amylases for survival [5]. Because these enzymes are active in the digestive tract and play a key role in carbohydrate metabolism, they are ideal targets for seed-based pest management approaches. Genetically modified (GM) legumes (i.e., cowpeas, peas, chickpeas, and azuki beans) expressing the α-amylase inhibitor 1 (αAI-1) from the common bean, *Phaseolus vulgaris* L., are resistant to several bruchid species under laboratory [6]–[9] and field conditions [10]. The deployment of GM legumes expressing other types of digestive enzyme inhibitors to control bruchids, such as plant protease inhibitors, has also been suggested [11]–[13]. Robust, reproducible, and efficient transformation procedures are available for many legumes species [1]. In addition, the combination of plant resistance factors together with biological control agents, especially hymenopteran parasitoids, can substantially increase the bruchid control provided by host-plant resistance alone [14]–[16]. If the strategy of combining a bruchid-resistant GM legume and biological control is to be effective and sustainable, the insecticidal trait expressed by the resistant crop must not adversely affect bruchid antagonists. A conceptual model describing how GM legume seeds expressing αAI-1 could harm the biological control service provided by parasitoids of bruchids has been developed by Lüthi et al. [17]. The model consists of five sequential steps and could be applied for protease inhibitor-expressing plants as well. In the first two steps, the model (i) characterizes the targeted digestive enzymes in the beneficial species and (ii) assesses the *in vitro* susceptibility to the plant resistance factor. The information required to satisfy these two steps of the model are not available for bruchid parasitoids. In the case of bruchid parasitoids, the physiological and biochemical aspects of their nutrition remain relatively unknown, and their susceptibility to αAI-1 has never been investigated.

In this study, we have characterized the α-amylase and protease activities in extracts of larvae and adult females of five common hymenopteran exoparasitoids of last instar larvae or pupae of bruchid pests. We then conducted *in vitro* experiments to assess the susceptibility of the exoparasitoid α-amylases to αAI-1 from *P. vulgaris*; for comparison, these biochemical assays also included extracts of three bruchid species. Finally, we used direct feeding assays to evaluate the effects of a commercial wheat α-amylase inhibitor and a serine protease inhibitor on females of two parasitoid species.

## Materials and Methods

### Insects

#### Bruchids

The following bruchids were obtained from C. Adler (Julius Kühn-Institut, Germany) and were maintained for several years on chickpea (*Cicer arietinum* L.) seeds (Kabuli type) at 24±2°C, 60±5% r.h., and complete darkness: *Acanthoscelides obtectus* (Say), *Callosobruchus chinensis* (L.), and *Callosobruchus maculatus* (F.) (Coleoptera: Chrysomelidae).

#### Parasitoids

Seeds infested with bruchids and parasitoids were sent to us by several investigators. *Heterospilus prosopidis* Viereck (Hymenoptera: Braconidae) parasitizing *C. chinensis* reared on Azuki bean [*Vigna angularis* (Willd.)] seeds were provided by M. Shimada (University of Tokyo, Japan). *Anisopteromalus calandrae* (Howard) (Hymenotpera: Pteromalidae) and *Lariophagus distinguendus* (Först.) (Hymenoptera: Pteromalidae) reared on wheat (*Triticum aestivum* L.) seeds infested with *Sitophilus granarius* (L.) (Coleoptera: Curculionidae) were obtained from J. Steidle (Hohenheim University, Germany). *Dinarmus basalis* (Rond.) (Hymenoptera: Pteromalidae) and *Eupelmus vuilleti* (Crw.) (Hymenoptera: Eupelmidae) on cowpea [Vigna unguiculata (L.) Walp.] seeds infested with *C. maculatus* were provided by J.P. Monge (Tours University, France). Upon arrival, seeds were kept in a climate chamber at 24±2°C, 60±5% r.h., and complete darkness. Emerging adults were then transferred to plastic containers (10.5 cm diameter, 15 cm high) containing last-instar larvae and/or pupae of *C. chinensis* in chickpea seeds and were reared in the laboratory for at least two generations before the start of the experiments.

Females of all species but *H. prosopodis* are synovigenic, i.e., they are born with immature eggs and must feed on their host to sustain egg production.

### Characterization of Digestive Enzymes in Parasitoids and Bruchids and *in vitro* Inhibitory Activity of Purified *P. vulgaris* αAI-1

#### Preparation of insect extracts

Fourth-instar larvae of all bruchid species and third-instar larvae and 1-week-old females of all parasitoid species from the rearing colonies were used for the *in vitro* characterization of digestive enzymes. Insects were homogenized in ice-cold 0.15 M NaCl (100 insects ml^−1^). The homogenates were centrifuged at 10,000 *g* for 5 min, and the supernatants were pooled and stored frozen at −20°C to obtain soluble protein extracts for determination of enzymatic activity. Total protein content was determined according to the method of Bradford [18] using bovine serum albumin (BSA) as the standard.

#### Characterization of α-amylase activity

A series of overlapping buffers was used to generate a pH range from 3 to 11. Reaction buffers were: 0.1 M citrate (pH 3–6), 0.1 M phosphate (pH 6–7), 0.1 M Tris-HCl (pH 7–9), and 0.1 M glycine-NaOH (pH 9–11). All buffers contained 0.15 M NaCl and 5 mM MgCl_2_. Assays were performed using a modified version of the Bernfeld assay [19]. The standard assay volume was 100 µl, which contained 5 µg of insect protein extract and potato starch as substrate added to a final concentration of 0.5% (w/v). After the samples had been incubated at 30°C for 45 min, the reaction was stopped by the addition of 100 µl of dinitrosalicylic acid (DNSA) reagent, followed by boiling for 5 min in a thermoblock (1102, SKS Bio-medical Instruments Ltd., Luton, England). Then, 1 ml of distilled water was added to the solution, which was mixed and left at room temperature for 15 min. Finally, the absorbance was read at 540 nm in a spectrophotometer, and α-amylase activity was expressed as mg of maltose liberated min^−1^ mg protein^−1^. A standard curve with maltose was constructed to calculate α-amylase activity.

The α-amylase activity of each bruchid and parasitoid extract was further characterized at its optimum pH in the presence of the specific inhibitors acarbose and wheat α-AI. Both inhibitors were incubated with the extracts at room temperature for 15 min before the substrate was added. The inhibitors were added in 10 µl of distilled water to a final concentration of 0.001% (w/v).

#### In vitro inhibitory activity of purified P. vulgaris αAI-1

The susceptibility of extracts of bruchid larvae and of parasitoid larvae and females to purified αAI-1 was determined by *in vitro* assay. αAI-1 purified from the seeds of *P. vulgaris* as described by Marshall and Lauda [20] was provided by T.J.V. Higgins (CSIRO, Australia). Previous studies have indicated that the two *Callosobruchus* species used in the current investigation are highly susceptible to αAI-1 [21] whereas the α-amylase activity of *A. obtectus* is not [22]. The α-amylase activity in the extract of each parasitoid and bruchid species was assayed at its optimum pH as described above. αAI-1 at concentrations ranging from 0 to 1 µg ml^−1^ was preincubated with the insect extracts for 15 min at room temperature before the substrate was added. Results were expressed as the percentage of α-amylase activity relative to that in the absence of the inhibitor.

#### Characterization of general proteolytic activity

For determination of general proteolytic activity in the bruchid *C. chinensis* and in the parasitoids *H. prosopidis*, *E. vuilleti*, and *A. calandrae*, a series of overlapping buffers (see section 2.2.2) was used to generate a pH range from 3 to 11. The standard assay volume was 100 µl, which contained 10 µg of insect protein extract and sulfanilamide-azocasein as substrate added to a final concentration of 0.5% (w/v). Samples were subsequently incubated overnight at 30°C. The reaction was stopped by the addition of 50 µl of ice-cold 10% TCA. Samples were then centrifuged at 10,000 *g* for 5 min, and 120 µl of the supernatant was added to 18 µl of 5 M NaOH. Finally, absorbance was measured at 450 nm using a SpectrafluorPlus plate reader (Tecan, Männedorf, Switzerland), and general proteolytic activity was expressed as Abs_450_ min^−1^ mg protein^−1^.

The proteolytic activity of the *C. chinensis* extract and of each parasitoid extract was further characterized at its optimum pH in the presence of the following class-specific protease inhibitors: the serine protease inhibitors SKTI (soybean Kunitz trypsin inhibitor) and PMSF (phenylmethylsulphonyl fluoride), the cysteine protease inhibitors E-64 (L-*trans-*epoxysuccinyl-leucylamido-(4-guanidino)-butane) and IAA (iodoacetamide), the aspartic protease inhibitor pepstatin-A, and the metalloprotease inhibitor EDTA (ethylenediaminetetraacetic acid); final assay concentrations were 1 mM, 10 µM, 10 µM, 1 mM, 10 µM, and 10 mM, respectively. Protease inhibitors were incubated at room temperature for 15 min before the substrate was added. All compounds were added in 10 µl of 0.15 M NaCl, except PMSF and pepstatin-A, which were added in 10 µl of DMSO (dimethyl sulfoxide). The doses tested were selected according to the concentrations recommended by Beynon and Salvesen [23].

All enzymatic assays were carried out in triplicate, and appropriate blanks were used to account for spontaneous breakdown of substrates. Chemicals were purchased from Sigma-Aldrich (St. Louis, MO, USA).

### Effect of Wheat α-AI and SKTI on Female Parasitoid Survival

Bioassays were conducted to test the effect of the commercial inhibitors wheat α-AI and SKTI on the survival of females of the parasitoids *A. calandrae* and *E. vuilleti*. α-Amylase and serine protease inhibitors are known to have detrimental effects on pests of stored products. The two parasitoid species were selected because they are synovigenic and belong to different hymenopteran families. Females of *A. calandrae* and *E. vuilleti* from the rearing colonies were collected after emergence (<24 h), individually kept in plastic boxes (2×2×1.5 cm), and provided with two 0.5-µl droplets of a 2 M sucrose solution containing different concentrations of either wheat α-AI or SKTI (0, 0.1, and 1%, w/v). In the bioassay with wheat α-AI, potato starch (20 µg ml^−1^) was added to the sucrose solution to stimulate α-amylase, whereas in the bioassay with SKTI, BSA (20 µg ml^−1^) was added to stimulate protease activity. Thirty females were tested per treatment. The food solutions were changed three times per week and were never completely consumed during this time period. Survival of the females was recorded daily. Bioassays were conducted in a climate chamber at 25±2°C, 70±5% RH, and 16:8 h L:D. Previous assays had shown that both parasitoids ingest the sucrose solution containing either of the two inhibitors (unpublished observations).

### Data Analysis

The survival response of female parasitoids to wheat α-AI or SKTI was analyzed using the Kaplan-Meier procedure and Logrank test. The α-level was set at 5%. Statistical analyses were conducted with the software package Statistica (Version 9, StatSoft Inc., Tulsa, OK, USA).

## Results

### Characterization of α-amylase Activity

We analyzed the optimum pH, the specific activity, and the effect of two specific α-amylase inhibitors on the hydrolysis of potato starch to characterize α-amylase activity in several bruchid and hymenopteran parasitoids (Table 1). α-Amylase activity was detected over a broad range of pH in both bruchids and parasitoids. In general, α-amylase activity was much higher in bruchids than in the parasitic wasps, and in females than in larvae of parasitoids. Hydrolysis of potato starch was greatest at pH 5.5 in all bruchid larvae and at pH 5 to 7 in parasitoid larvae and females. Hydrolysis of potato starch was greatly inhibited by acarbose and wheat αAI in extracts from all insects.

**Table 1:** α-Amylase activity (mg of maltose liberated min^−1^ mg^−1^ protein) and inhibition of potato starch hydrolysis by the specific inhibitors acarbose and wheat αAI in extracts of bruchids and hymenopteran parasitoids.

Source of extract			α-Amylase activity		Inhibition (%)^c^	
Family	Species	Stage^a^	Optimum pH	Specific activity^b^	Acarbose (10^−3%^)	Wheat αAI (10^−3%^)
Chrysomelidae	*A. obtectus*	L	5.5	0.68±0.010	98.9±0.55	83.9±1.43
	*C. chinensis*	L	5.5	0.84±0.023	98.0±1.09	51.2± 3.31
	*C. maculatus*	L	5.5	0.51±0.032	97.2±0.33	91.4±0.17
Braconidae	*H. prosopidis*	L	6.0	0.07±0.006	35.0±1.77	71.7±0.43
		F	6.0	0.14±0.001	97.9±0.17	24.8±1.78
Eupelmidae	*E. vuilleti*	L	6.0	0.17±0.000	77.8±0.26	73.0±1.31
		F	6.0	0.25±0.003	95.6±0.61	40.4±4.13
Pteromalidae	*A. calandrae*	L	5.0	0.02±0.000	99.2±0.20	86.1±1.96
		F	6.0	0.81±0.023	68.2±0.80	87.4±2.77
	*D. basalis*	L	5.0	0.21±0.001	60.4±3.37	63.7±4.21
		F	6.0	0.38±0.017	94.4±0.30	49.6±1.08
	*L. distinguendus*	L	5.0	0.04±0.003	96.2±0.80	82.2±0.00
		F	7.0	0.84±0.032	61.6±1.91	75.9±4.57

a L  =  larvae; F  =  females.

b Values are means ± SE of triplicate measurements for a unique pool of extracts.

c The percentage of inhibition was calculated as [1-(activity with an inhibitor/activity in control) ×100]. Inhibition was measured at the optimum pH.

### *In vitro* Inhibitory Activity of Purified *Phaseolus Vulgaris* αAI-1

**Figure 1:**
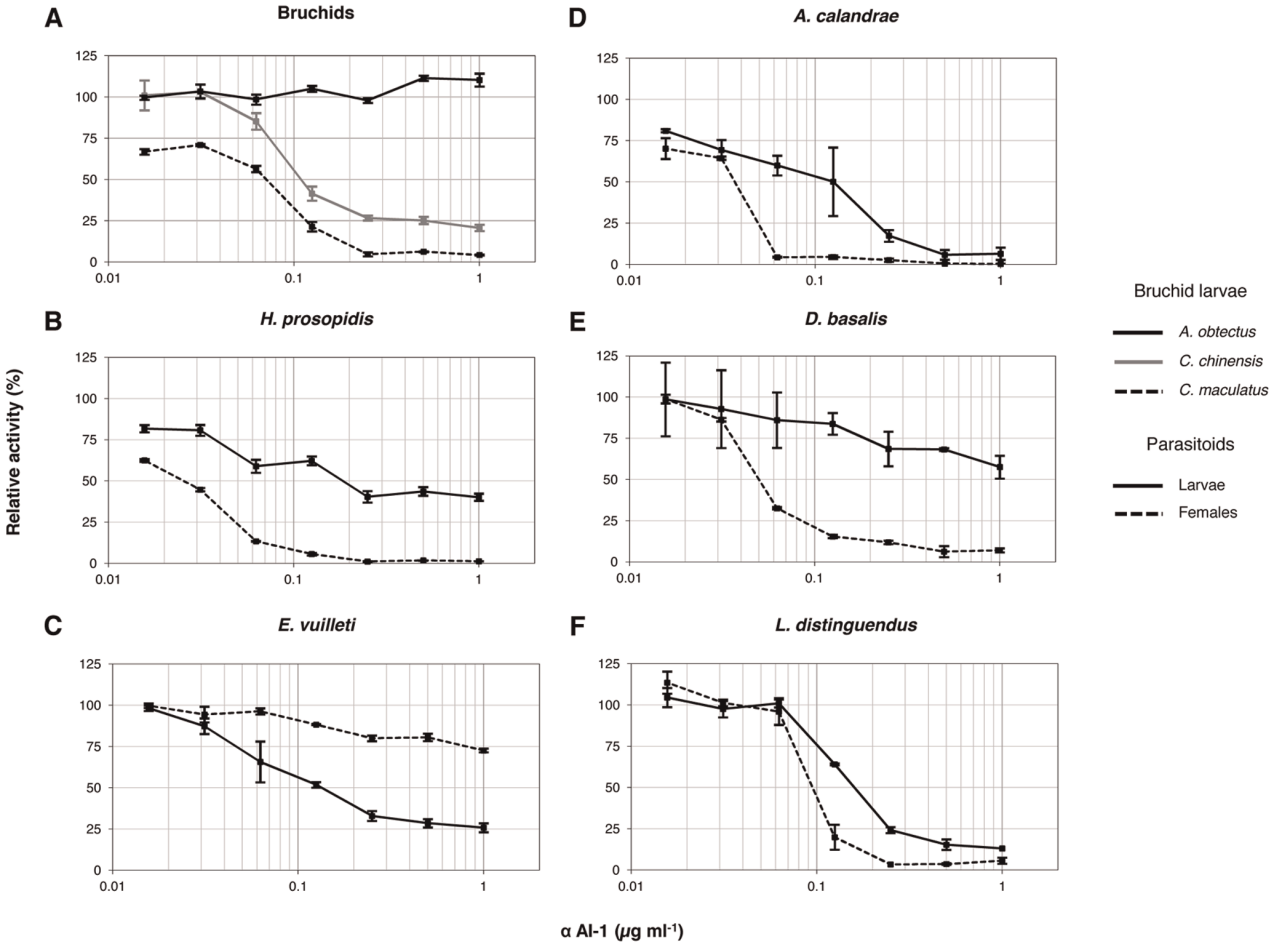
*In vitro* activity of αAI-1 from *Phaseolus vulgaris*. Inhibitory activity of αAI-1 against α-amylase activity in extracts of (A) three bruchids (*Acanthoscelides obtectus*, *Callosobruchus chinensis*, and *Callosobruchus maculatus*) and in extracts of larvae and adult females of the hymenopteran parasitoids (B) *Heterospilus prosopidis*, (C) *Eupelmus vuilleti*, (D) *Anisopteromalus calandrae*, (E) *Dinarmus basalis*, and (F) *Lariophagus distinguendus*. Relative activity was calculated as [(activity with αAI-1/activity without αAI-1)×100]. Bars represent means ± SE of three measurements from a unique pool of extracts.

*In vitro* inhibition studies were conducted to assess the susceptibility of bruchids and parasitoids to purified αAI-1. α-Amylase activity in extracts from all parasitoid larvae and females was reduced by the inhibitor (Fig. 1B–F). Except for *E. vuilleti* (Fig. 1C), inhibition was greater in females than in larvae of parasitoids. α-Amylase inhibition caused by αAI-1 in the susceptible bruchid species *C. chinensis* and *C. maculatus* was similar to that in the hymenopteran parasitoids (Fig. 1A). As expected, the α-amylase activity of *A. obtectus* was not inhibited by αAI-1 (Fig. 1A).

### Characterization of General Proteolytic Activity

We characterized proteolytic activity in protein extracts of the bruchid *C. chinensis* and the parasitoids *H. prosopidis*, *E. vuilleti*, and *A. calandrae*. Hydrolysis of the general substrate azocasein occurred over a broad range of pH in all species, although the profile differed for *C. chinensis* vs. the three parasitoids (Table 2). While azocasein was hydrolyzed mainly at acidic pH values by the bruchid, and bruchid proteolytic activity was highest at pH 5.0, proteolysis in parasitoid larvae and females was highest at alkaline pH values. Proteolytic activities were further characterized with specific diagnostic protease inhibitors (Table 2). Proteolytic activity of *C. chinensis* was inhibited by the cysteine-like protease inhibitors E-64 and IAA, and by the aspartic protease inhibitor pepstatin-A. In contrast, general proteolysis was inhibited by the serine proteases inhibitors SKTI and PMSF in larvae of the parasitoids *H. prosopidis* and *A. calandrae,* and only by SKTI in *E. vuilleti* larvae. Hydrolysis of azocasein was reduced by SKTI and the metalloproteases inhibitor EDTA in females of *A. calandrae* and *E. vuilleti*. Together, these results indicate that the proteolytic mechanisms differ for the bruchid *C. chinensis* and the three parasitoids: whereas *C. chinensis* relies on cysteine and aspartic proteases, parasitoid larvae rely on serine proteases, and parasitoid females rely on serine proteases and metalloproteases.

**Table 2:** Proteolytic activity (as indicated by Abs_450_ min^−1^ mg protein^−1^) and inhibition of azocasein hydrolysis by specific protease inhibitors in extracts of *Callosobruchus chinensis* and three parasitoid species.

Source of extract			Proteolytic activity		Inhibition (%)^c^					
Family	Species	Stage^a^	Optimum pH	Specific activity^b^	PMSF (10 mM)	SKTI (10 µM)	E-64 (10 µM)	IAA (1 mM)	EDTA (10 mM)	Pepstatin-A (10 µM)
Chrysomelidae	*C. chinensis*	L	5.0	22.2±0.12	ni	ni	68.2±1.03	52.6±0.39	ni	34.9±2.56
Braconidae	*H. prosopidis*	L	9.0	216.4±17.56	24.4±4.18	75.4±2.78	ni	ni	ni	ni
		F	7.0	3.1±0.55	nd	nd	nd	nd	nd	nd
Eupelmidae	*E. vuilleti*	L	10.0	196.7±1.15	ni	83.1±1.20	ni	ni	ni	ni
		F	8.0	31.0±1.56	ni	78.6±0.89	ni	ni	40.6±3.95	ni
Pteromalidae	*A. calandrae*	L	9.0	147.2±9.88	36.3±4.18	75.4±2.78	ni	ni	ni	ni
		F	8.0	144.2±4.10	ni	69.3±1.24	ni	ni	86.5±0.98	ni

a L  =  larvae; F  =  females.

b Values are means ± SE of triplicate measurements for a unique pool of extracts.

c The percentage of inhibition was calculated as [1-(activity with an inhibitor/activity in control) ×100]. Inhibition was measured at the optimum pH.

Protease inhibitors abbreviations: PMSF (phenylmethylsulphonyl fluoride), SKTI (soybean Kunitz trypsin inhibitor), E-64 (L-*trans-*epoxysuccinyl-leucylamido-(4-guanidino)-butane), IAA (iodoacetamide), EDTA (ethylenediaminetetraacetic acid).

“ni”: inhibition <10%.

“nd”: not determine.

### Effect of Two Digestive Inhibitors, Wheat αAI and SKTI, on Female Parasitoid Survival

**Figure 2:**
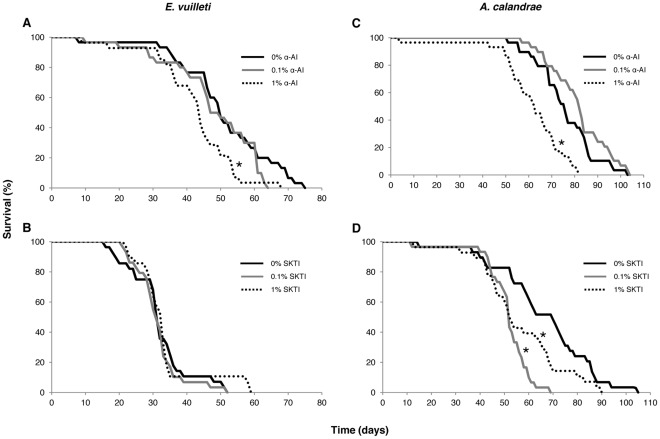
Effect of wheat αAI and SKTI on bruchid parasitoids. Survival of females of the parasitoids *Eupelmus vuilleti* (2A, 2B) and (2C, 2D) *Anisopteromalus calandrae* fed with a 2 M sucrose solution containing different amounts of either wheat αAI or the serine protease inhibitor SKTI. (*N* = 28−30). Potato starch or BSA (20 µg ml^−1^) was added to the sucrose solution to stimulate α-amylase and protease activity, respectively. An asterisk indicates that survival was significantly lower with wheat αAI or SKTI than with the control (Logrank test, *P*<0.05).

The effect of wheat α-AI and the serine protease inhibitor SKTI on the survival of *E. vuilleti* and *A. calandrae* females was investigated *in vivo*. For *E. vuilleti*, female longevity in the control treatments was 51.0±2.64 days (mean ± SE) in the wheat α-AI assay and 31.9±1.67 days in the SKTI assay. For *A. calandrae*, female longevity in the control treatments was 75.8±2.32 days in the wheat α-AI assay and 66.2±3.65 days in the SKTI assay. The survival of *E. vuillleti* and *A. calandrae* fed with 1% wheat α-AI was significantly lower than in the control (*P* = 0.007 and *P* = 0.035, respectively) (Fig. 2A, 2C). SKTI significantly decreased the survival of *A. calandrae* at 0.1% and 1% concentration (*P*<0.001 and *P* = 0.044, respectively) (Fig. 2D) whereas no effect was observed in *E. vuilleti* females (Fig. 2B).

## Discussion

### Characterization of α-amylase Activity

α-Amylase activity has been mainly studied in lepidopteran and coleopteran (including bruchids) storage pests. Only a few studies have characterized the α-amylase activity in insect predators [24]–[27] and parasitoids [28], and to our knowledge α-amylase activity has never been characterized in any parasitoid of bruchids. In our study, significant α-amylase activity was measured in extracts of all parasitoid species tested. Hydrolysis of potato starch was highest at pH 5–6 for larval extracts and at pH 6–7 for female extracts. Similar pH values for optimal α-amylase activity were recorded by Kluh et al. [28] for three hymenopteran species that included adults of the ichneumonid *Venturia canescens* Grav., which is an endoparasitoid of lepidopteran larvae. As previously reported by Podoler and Applebaum [29], Campos et al. [30], and Franco et al. [31], α-amylase activity in the bruchid larvae in the current study was optimal under acidic conditions. α-Amylase activity in parasitoid extracts was usually greater in females than in larvae. This difference might be due to the ability of female parasitoids to feed on carbohydrate-rich sources, like floral and extra-floral nectar [32]. Except for extracts from *A. calandrae* and *L. distinguendus* females, α-amylase activity was lower in parasitic wasp extracts than in bruchid larval extracts. This is not surprising because bruchid larvae exclusively feed on starchy legume seeds and therefore rely mainly on α-amylases for food digestion.

### *In vitro* Inhibitory Activity of Purified *Phaseolus Vulgaris* αAI-1

In our study, the α-amylase activity of all hymenopteran parasitoids was reduced when exposed to purified αAI-1. With the exception of *E. vuilleti*, *in vitro* inhibition was higher in females than in larvae. For comparison, larval extracts of the bruchids *A. obtectus*, *C. chinensis*, and *C. maculatus* were also exposed to purified αAI-1. The α-amylase activity of *C. chinensis* and *C. maculatus* larvae was reduced by αAI-1, and the reduction was greater for *C. maculatus*. This is in agreement with previous reports that neither species was able to grow on seeds of the common bean [33], [34]. In contrast, the α-amylase activity in larval extracts of *A. obtectus* was insensitive to αAI-1. The reasons for which *A. obtectus* can survive on common beans remain unknown [35]. Our *in vitro* results with larvae of the three bruchid species are in agreement with those published by Ishimoto and Kitamura [21], [22]. Our results also revealed that the inhibition of α-amylase activity by αAI-1 in the parasitoids was similar to that in their bruchid hosts.

The inhibitor αAI-1 has been shown to have a broad spectrum of *in vitro* activity against arthropods. Kluh et al. [28] screened the *in vitro* inhibitory activity of purified αAI-1 against α-amylases from 30 arthropod species representing nine orders. The α-amylases from coleopteran, dipteran, and hymenopteran species, including that from the parasitoid *V. canescens*, were susceptible to the inhibitor. Together with our results, it appears that coleopteran and hymenopteran species are highly sensitive to αAI-1 in general. In contrast, α-amylase activities of lepidopteran larvae as well as of those species belonging to Blattodea, Psocoptera, Orthoptera, Hemiptera, and Acari were not inhibited by αAI-1 at concentrations up to 1 µM [28].

### Characterization of General Proteolytic Activity

We partially characterized the proteolytic enzymes in larval and female extracts of the parasitic wasps *H. prosopidis*, *E. vuilleti*, and *A. calandrae*, and in larval extracts of the bruchid *C. chinensis*. General proteolytic activity in the parasitic wasps was highest under alkaline conditions and higher in larvae than in females. This might be due to different nutritional requirements of females vs. larvae: while females consume the small amounts of haemolymph that exude from punctures in the host cuticle [36], the larvae consume most of their host. The ability of extracts to hydrolyze the general substrate azocasein, the optimal pH for hydrolysis, and the sensitivity to a range of protease inhibitors demonstrated that larvae predominantly rely on serine proteases for protein digestion while *E. vuilleti* and *A. calandrae* females contain both serine proteases and metalloproteases. Because females are host-feeders, they feed from the same resource as the larvae. Such serine-protease based digestive metabolism has been previously reported from other hymenopteran species, including ants [37], bees [38], bumblebees [39], and parasitic wasps. Trypsin- and chymotrypsin-like activities were detected in larvae of the ectoparasitoid *Eulophus pennicornis* (Nees) (Hymenoptera: Eulophidae) [40]. Similarly, the predominant proteases detected in larvae of the ectoparasitoid *Habrobracon hebetor* Say (Hymenoptera: Braconidae) and in larvae and adults of the aphid parasitoids *Aphelinus abdominalis* Dalman (Hymenoptera: Aphelinidae) and *Aphidius ervi* Haliday (Hymenoptera: Braconidae) belonged to the serine protease class [41]–[43]. In contrast to parasitic wasps, maximal hydrolysis of azocaseine in larval extracts of the bruchid *C. chinensis* occurred at acidic pH. Indeed, the proteolytic enzymes detected differed between *C. chinensis* larvae and their hymenopteran parasitoids in that both cysteine and aspartic proteases were detected in *C. chinensis* larvae but not in the parasitoids. Our results agree with those of similar studies conducted with several bruchid species, including *C. chinensis* [44]–[47].

### Effect of Digestive Inhibitors (Wheat αAI and SKTI) on Female Parasitoid Survival

The two parasitoids tested in our study differed in susceptibility to digestive enzyme inhibitors. The survival of the *E. vuilleti* females was reduced by wheat αAI, while the survival of *A. calandrae* was reduced by wheat αAI and the serine protease inhibitor SKTI. Although serine protease activity in extracts of *E. vuilleti* was inhibited *in vitro* by SKTI, no effect was observed when the protease inhibitor was administered *in vivo*. This could be explained by the ability of some natural enemies to adapt their digestive metabolism to the presence of plant antidigestive proteins [48].

The negative effects of the test compounds on both parasitoids are unlikely to be biologically relevant because females that fed on αAI and SKTI survived much longer than required for oviposition. Survival of susceptible females that consumed αAI and SKTI was not affected until after day 50, but in previous studies, female *A. calandrae* and *E. vuilleti* kept on seeds infested with bruchid larvae, and thus able to oviposit, lived for only 10 and 13 days, respectively [14], [49]. Only a few studies have assessed the *in vivo* effects of serine protease inhibitors on hymenopteran parasitoids, and the impact of digestive enzyme inhibitors on bruchid parasitoids has never been previously evaluated. Negative, host-mediated effects of the cowpea trypsin inhibitor (CpTI) and the soybean Bowman-Birk inhibitor (SbBBI) on adult *E. pennicornis* and *A. abdominalis* were reported by Bell et al. [50] and Azzouz et al. [42], respectively. When those parasitoid species were directly fed with sugar solutions containing the protease inhibitor, however, no detrimental effects were observed, suggesting that the negative effects were due to reduced host quality (because the hosts are known to be susceptible to the test compound) rather than to a direct effect of the inhibitor.

### Conclusions

This is the first study to characterize the α-amylase and proteolytic enzymes in parasitoids of bruchids and to assess the *in vitro* susceptibility of the parasitoids to αAI-1. The results of our study also provide information required by the model of Lüthi et al. [17], which assesses the potential effects of GM legumes on non-target species.

The results of this study indicate that serine proteases are the main digestive enzymes in all the parasitoids of bruchids that were tested, whereas cysteine proteases are the main digestive enzymes in bruchid larvae. It follows that the use of GM legumes expressing cysteine protease inhibitors to control bruchids should be compatible with the control provided by hymenopteran parasitoids. We have also detected significant levels of α-amylase activity in larval and female extracts of several hymenopteran parasitoid species used for bruchid control. Subsequent *in vitro* inhibition studies showed that sensitivity of α-amylase activity to αAI-1 in the bruchid antagonists was comparable to that in the target species, suggesting that parasitoids might be negatively affected by αAI-1-expressing legumes if they consume this inhibitor when feeding on their hosts. Direct feeding assays performed in our study under worst-case exposure conditions revealed that harmful effects of αAI on bruchid parasitoids cannot be discounted. Future bioassays should therefore be conducted under more realistic exposure conditions to evaluate the compatibility of αAI-1-expressing GM legumes with biological control agents for bruchid management.

## References

[1] Popelka JC, Terryn N, Higgins TJV (2004). Gene technology for grain legumes: Can it contribute to the food challenge in developing countries?. Plant Sci.

[2] Nukenine EN (2010). Stored product protection in Africa: Past, present and future.. Julius-Kühn-Archiv.

[3] Odogola WR (1994). Postharvest management and storage of food legumes..

[4] Philips TW, Throne JE (2010). Biorational approaches to managing stored-product insects.. Ann Rev Entomol.

[5] Franco OL, Rigden DJ, Melo FR, Grossi-de-Sá MF (2002). Plant α-amylase inhibitors and their interaction with insect α-amylases. Structure, function and potential for crop protection.. Eur J Biochem.

[6] Schroeder HE, Gollasch S, Moore A, Tabe LM, Craig S (1995). Bean α-amylase inhibitor confers resistance to the pea weevil (*Bruchus pisorum*) in transgenic peas (*Pisum sativum* L.).. Plant Physiol.

[7] Ishimoto M, Sato T, Chrispeels MJ, Kitamura K (1996). Bruchid resistance of transgenic azuki bean expressing seed α-amylase inhibitor of common bean.. Entomol Exp Appl.

[8] Sarmah BK, Moore A, Tate W, Molvig L, Morton RL (2004). Transgenic chickpea seeds expressing high levels of a bean α-amylase inhibitor.. Mol Breeding.

[9] Solleti SK, Bakshi S, Purkayasthan J, Panda SK, Sahoo L (2008). Transgenic cowpea (*Vigna unguiculata*) seeds expressing a bean α-amylase inhibitor 1 confer resistance to storage pests, bruchid beetles.. Plant Cell Rep.

[10] Morton RL, Schroeder HE, Bateman KS, Chrispeels MJ, Armstrong E (2000). Bean alpha amylase inhibitor 1 in transgenic peas (*Pisum sativum*) provides complete protection from pea weevil (*Bruchus pisorum*) under field conditions.. Proc Natl Acad Sci USA.

[11] Koiwa H, Shade RE, Zhu-Salzman K, Subramanian L, Murdock LL (1998). Phage display selection can differentiate insecticidal activity of soybean cystatins.. Plant J.

[12] Amirhusin B, Shade RE, Koiwa H, Hasegawa PM, Bressan RA (2004). Soyacystatin N inhibits proteolysis of wheat α-amylase inhibitor and potentiates toxicity against cowpea weevil.. J Econ Entomol.

[13] Amirhusin B, Shade RE, Koiwa H, Hasegawa PM, Bressan RA (2007). Protease inhibitors from several classes work synergistically against *Callosobruchus maculatus*.. J Insect Physiol.

[14] Schmale I, Wäckers FL, Cardona C, Dorn S (2001). Control potential of three hymenopteran parasitoid species against the bean weevil in stored beans: the effect of adult parasitoid nutrition on longevity and progeny production.. Biol Control.

[15] Schmale I, Wäckers FL, Cardona C, Dorn S (2003). Combining parasitoids and plant resistance for the control of the bruchid *Acanthoscelides obtectus* in stored beans.. J Stored Prod Res.

[16] Velten G, Rott AS, Conde Petit BJ, Cardona C, Dorn S (2008). Improved bruchid management through favorable host plant traits and natural enemies.. Biol Control.

[17] Lüthi C, Álvarez-Alfageme F, Romeis J (2010). The potential of transgenic legumes in integrated bruchid management: assessing the impact on bruchid parasitoids.. Julius-Kühn-Archiv.

[18] Bradford MM (1976). A rapid and sensitive method for the quantitation of microgram quantities of protein utilizing the principle of protein-dye binding.. Anal Biochem.

[19] Bernfeld P (1955). Amylases, α and β.. Methods Enzymol.

[20] Marshall JJ, Lauda CM (1975). Purification and properties of phaseolamin, an inhibitor of α-amylase, from kidney bean, *Phaseolus vulgaris*.. J Biol Chem.

[21] Ishimoto M, Kitamura K (1989). Growth inhibitory effects of α-amylase inhibitor from the kidney bean, *Phaseolus vulgaris* (L) on 3 species of bruchids (Coleoptera, Bruchidae).. Appl Entomol Zool.

[22] Ishimoto M, Kitamura K (1992). Tolerance to the seed α-amylase inhibitor by the two insect pests of the common bean, *Zabrotes subfasciatus* and *Acanthoscelides obtectus* (Coleoptera, Bruchidae).. Appl Entomol Zool.

[23] Beynon RJ, Salvesen G (1989). Proteolytic enzymes: a practical approach.. In: Beynon RJ, Bond JS (Eds.) IRL Press at Oxford Inoversoty Press, Oxford,.

[24] Ferreira C, Terra WR (1989). Spatial organization of digestion, secretory mechanisms and digestive enzyme properties in *Pheropsophus aequinoctialis* (Coleoptera, Carabidae).. Insect Biochem.

[25] Zeng F, Cohen AC (2000). Demonstration of amylase from the zoophytophagous anthocorid *Orius insidiosus*.. Arch Insect Biochem.

[26] Boyd DW, Cohen AC, Alverson DR (2002). Digestive enzymes and stylet morphology of *Deraeocoris nebulosis* (Hemiptera: Miridae), a predacious plant bug.. Ann Entomol Soc Am.

[27] Oliveira JA, Oliveira MG, Guedes RN, Soares MJ (2006). Morphology and preliminary enzyme characterization of the salivary glands from the predatory bug *Podisus nigrispinus* (Heteroptera: Pentatomidae).. Bull Entomol Res.

[28] Kluh I, Horn M, Hýblová J, Hubert J, Dolečková-Marešová L (2005). Inhibitory specificity and insecticidal selectivity of α-amylase inhibitor from *Phaseolus vulgaris*.. Phytochemistry.

[29] Podoler H, Applebau SW (1971). Alpha-amylase of beetle *Callosobruchus chinensis* – Properties.. Biochem J.

[30] Campos FAP, Xavier J, Silva CP, Ary MB (1989). Resolution and partial characterization of proteinases and alpha-amylases from midguts of larvae of the bruchid beetle *Callosobruchus maculatus* (F.).. Comp Biochem Phys B.

[31] Franco OL, Melo FR, Mendes PA, Paes NS, Yokoyama M (2005). Characterization of two *Acanthoscelides obtectus* α-amylases and their inactivation by wheat inhibitors.. J Agr Food Chem.

[32] Jervis MA, Kidd NAC, Heimpel GE (1996). Parasitoid adult feeding behaviour and biocontrol – a review.. Biocontrol News and Information.

[33] Ishii S (1952). Studies on host preference of the cowpea weevil (*Callosobruchus chinensis* L.).. Bull Natl Inst Agr Sci Ser.

[34] Janzen DH, Juster HB, Liener IE (1976). Insecticidal action of the phytohemagglutinin in black beans on a bruchid beetle.. Science.

[35] Ishimoto M, Chrispeels MJ (1996). Protective mechanism of the Mexican bean weevil against high levels of alpha-amylase inhibitor in the common bean.. Plant Physiol.

[36] Jervis MA, Kidd NAC (1986). Host-feeding strategies in hymenopteran parasitoids.. Biol Rev.

[37] Whitworth ST, Blum MS, Travis J (1998). Proteolytic enzymes from larvae of the fire ant, *Solenopsis invicta*: Isolation and characterization of four serine endopeptidases.. J Biol Chem.

[38] Moritz B, Crailsheim K (1987). Physiology of protein digestion in the midgut of the honeybee (*Apis mellifera* L.).. J Insect Physiol.

[39] Malone LA, Burgess EPJ, Stefanovic D, Gatehouse HS (2000). Effects of four protease inhibitors on the survival of worker bumblebees, *Bombus terrestris* L. Apidologie.

[40] Down RE, Ford L, Mosson HJ, Fitches E, Gatehouse J (1999). Protease activity in the larval stage of the parasitoid wasp, *Eulophus pennicornis* (Nees) (Hymenoptera: Eulophidae); effects of protease inhibitors.. Parasitology.

[41] Baker JE, Fabrick JA (2000). Host hemolymph proteins and protein digestion in larval *Habrobracon hebetor* (Hymenoptera: Braconidae).. Insect Biochem Molec.

[42] Azzouz H, Campan EDM, Cherqui A, Saguez J, Couty A (2005). Potential effects of plant protease inhibitors, oryzacystatin I and soybean Bowman-Birk inhibitor, on the aphid parasitoid *Aphidius ervi* Haliday (Hymenoptera, Braconidae).. J Insect Physiol.

[43] Azzouz H, Cherqui A, Campan EDM, Rahbe Y, Duport G (2005). Effects of plant protease inhibitors, oryzacystatin I and soybean Bowman-Birk inhibitor, on the aphid *Macrosiphum euphorbiae* (Homoptera, Aphididae) and its parasitoid *Aphelinus abdominalis* (Hymenoptera, Aphelinidae).. J Insect Physiol.

[44] Kitch LW, Murdock LL (1986). Partial characterization of a major gut thiol proteinase from larvae of *Callosobruchus maculatus* F. Arch Insect Biochem.

[45] Silva CP, Xavier-Filho J (1991). Comparison between the levels of aspartic and cysteine proteinases of the larval midguts of *Callosobruchus maculatus* (F) and *Zabrotes subfasciatus* (Boh) (Coleoptera, Bruchidae).. Comp Biochem Physiol B.

[46] Kuroda M, Ishimoto M, Suzuki K, Kondo H, Abe K (1996). Oryzacystatins exhibit growth-inhibitory and lethal effects on different species of bean insect pests, *Callosobruchus chinensis* (Coleoptera) and *Riptortus clavatus* (Hemiptera).. Biosci Biotech Biochem.

[47] Melo FR, Mello MO, Franco OL, Rigden DJ, Mello LV (2003). Use of phage display to select novel cystatins specific for *Acanthoscelides obtectus* cysteine proteinases.. BBA-Proteins Proteom.

[48] Álvarez-Alfageme F, Martinez M, Pascual-Ruiz S, Castanera P, Diaz I (2007). Effects of potato plants expressing a barley cystatin on the predatory bug *Podisus maculiventris* via herbivorous prey feeding on the plant.. Transgenic Res.

[49] Giron D, Pincebourde S, Casas J (2004). Lifetime gains of host-feeding in a synovigenic parasitic wasp.. Physiol Entomol.

[50] Bell HA, Kirkbride-Smith AE, Marris GC, Edwards JP, Gatehouse AMR (2004). Oral toxicity and impact on fecundity of three insecticidal proteins on the gregarious ectoparasitoid *Eulophus pennicornis* (Hymenoptera: Eulophidae).. Agr Forest Entomol.

